# UNADON: transformer-based model to predict genome-wide chromosome spatial position

**DOI:** 10.1093/bioinformatics/btad246

**Published:** 2023-06-30

**Authors:** Muyu Yang, Jian Ma

**Affiliations:** Computational Biology Department, School of Computer Science, Carnegie Mellon University, Pittsburgh PA 15213, USA; Computational Biology Department, School of Computer Science, Carnegie Mellon University, Pittsburgh PA 15213, USA

## Abstract

**Motivation:**

The spatial positioning of chromosomes relative to functional nuclear bodies is intertwined with genome functions such as transcription. However, the sequence patterns and epigenomic features that collectively influence chromatin spatial positioning in a genome-wide manner are not well understood.

**Results:**

Here, we develop a new transformer-based deep learning model called UNADON, which predicts the genome-wide cytological distance to a specific type of nuclear body, as measured by TSA-seq, using both sequence features and epigenomic signals. Evaluations of UNADON in four cell lines (K562, H1, HFFc6, HCT116) show high accuracy in predicting chromatin spatial positioning to nuclear bodies when trained on a single cell line. UNADON also performed well in an unseen cell type. Importantly, we reveal potential sequence and epigenomic factors that affect large-scale chromatin compartmentalization in nuclear bodies. Together, UNADON provides new insights into the principles between sequence features and large-scale chromatin spatial localization, which has important implications for understanding nuclear structure and function.

**Availability and implementation:**

The source code of UNADON can be found at https://github.com/ma-compbio/UNADON.

## 1 Introduction

The cell nucleus of higher eukaryotes is a heterogeneous organelle that contains distinct subnuclear structures and nuclear bodies associated with crucial cellular functions (Spector 2001, Dundr and Misteli 2010). For example, nuclear speckles regulate the transcription and splicing of genes (Spector 2001, Chen and Belmont 2019) and the nuclear lamina anchors the chromatin toward the nuclear periphery (Van Steensel and Belmont 2017). Chromosomes are folded and packaged inside the nucleus, and they interact with various types of nuclear bodies, which has important implications for the structure and function of the higher-order genome organization. In particular, the gene expression level is found to be inversely correlated with distance to nuclear speckles (Zhang et al. 2020), and nuclear lamina frequently interacts with repressive chromatin (Van Steensel and Belmont 2017). In the past decade, the rapid development of genome-wide mapping of chromatin interactions such as Hi-C (Lieberman-Aiden et al. 2009) has revealed that the nuclear genome has multiscale structures, including A/B compartments (Lieberman-Aiden et al. 2009) and subcompartments (Rao et al. 2014), topologically associating domains (TADs) (Dixon et al. 2012), and chromatin loops (Jin et al. 2013, Rao et al. 2014). However, the interactions between large-scale chromatin and functional nuclear bodies in the nucleus remain poorly understood. The recently developed tyramide signal amplification sequencing (TSA-seq) genomic mapping method (Chen et al. 2018) measures the cytological distances from chromosome loci to nuclear bodies, providing unprecedented opportunities to probe the spatial positioning of chromosomes relative to specific nuclear bodies in a genome-wide manner. Recent work has integrated different 3D genome mapping data to generate models of genome organization, including their distances to nuclear landmarks (Boninsegna et al. 2022). To reach a more complete understanding of the interplay between nuclear structure and function, it is imperative to reveal the underlying principles and determinants (e.g., sequence features and various local epigenomic signals) for chromatin-nuclear body interactions in a wide range of cellular contexts. Unfortunately, there are currently no computational methods available to facilitate such analysis by combining genome sequence features and other genome-wide epigenomic features.

In the past few years, deep learning models have demonstrated strong prediction performance for functional genomic and epigenomic features (Eraslan et al. 2019, Yang and Ma 2022). Specifically, deep learning architectures have been applied to predict 3D genome features in different scales based on genomic sequence features (Yang and Ma 2022). Computational methods such as Akita (Fudenberg et al. 2020) integrate DNA sequences up to 1 Mb by using a series of dilated convolutional neural network (CNN) layers to expand the receptive field. However, this approach may miss important dependencies for spatial positioning domains that can be as large as 10 Mb (Briand and Collas 2020). A more recent method Orca (Zhou 2022) expands the maximum input sequence length to the whole chromosome by using a large CNN with a cascading prediction mechanism. However, the ability of CNN-based models to capture long-range dependencies is restricted by the size of the receptive field. Deeper CNNs with larger dilation rates are required for large-scale chromatin feature predictions with long sequences, which is computationally inefficient to train. Besides, very few existing models combine both genomic sequence and epigenomic features to effectively perform cross-cell-type predictions.

Here, we introduce a new multi-modal transformer-based deep learning model called UNADON, which predicts chromatin spatial positioning relative to nuclear bodies based on DNA sequences and epigenomic signals. The major contributions of our work are as follows: (i) UNADON is a deep learning-based model to specifically predict chromatin spatial positioning relative to nuclear bodies; (ii) The distinctive neural architecture design enables UNADON to learn the long-range dependencies more effectively; (iii) UNADON generalizes well in the cross-cell-type predictions, which can be applied to infer spatial positioning in new cell types. (iv) Interpretation of UNADON reveals potential mechanisms for targeting nuclear bodies. Together, UNADON establishes a novel computational framework for the prediction and interpretation of the spatial positioning of chromosomes.

## 2 Materials and methods

### 2.1 Overview of UNADON

UNADON is a transformer-based deep learning framework for predicting the spatial positioning of chromosomes relative to nuclear bodies from DNA sequence features and epigenomic signals. To integrate the input data of different modalities and enhance model interpretability, we highlight the following three distinctive features of UNADON: (i) preprocessing of the sequence and epigenomic signals enables efficient learning of complex features; (ii) the multi-modal design allows the network to extract features for sequence and epigenomic signals separately; (iii) the transformer modules capture crucial long-range dependencies of genomic bins.

**Figure 1.**
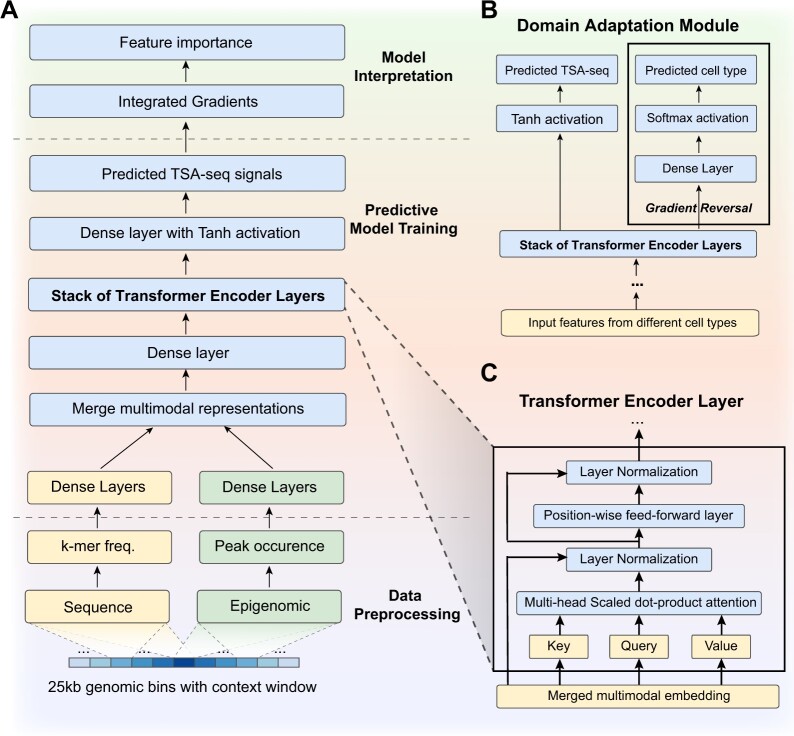
(A) Overview of UNADON. The workflow consists of three steps. (i) Data preprocessing. For each genomic region of 25 kb, the DNA sequence feature is represented by a vector of PCA-reduced k-mer frequency. The epigenomic feature is represented by the occurrence of epigenomic signal peaks within the region. (ii) Predictive model training. To effectively incorporate different data modalities and large-context information, the model adopts a multi-modal design and uses the transformer modules with self-attention mechanisms. (iii) Model interpretation. We evaluate the important sequence elements and epigenomic signals using the feature contribution score computed by integrated gradients. (B) An illustration of the domain adaptation techniques applied on top of UNADON in the cross-cell-type predictions. (C) The details of the transformer encoder layers.

The workflow of UNADON comprises three components: data preprocessing, predictive model training, and model interpretation (see Fig. 1A for an overview). The genome is segmented into non-overlapping bins as the basic units for prediction and interpretation. The model considers all genomic bins within a certain window size as input to provide context information. The processed DNA and epigenomic features are fed into two subnetworks consisting of dense layers to learn the sequence and epigenomic representations independently, which are merged and passed into the transformer encoder modules. The most important component of the transformer encoder modules is the multi-head attention layers. The attention layers automatically learn the relevance of the neighboring bins in predicting the TSA-seq signal, allowing the model to incorporate long-range information. After training and evaluation, a post-hoc feature attribution method, integrated gradients, is applied to systematically estimate the importance of loci and the contribution of different features.

### 2.2 Neural network architecture in UNADON

The network architecture for UNADON is shown in Fig. 1A. The details of the architecture are as follows. For predicting the TSA-seq signal for one 25 kb genomic bin, we consider the large context of ±2.5 Mb. The pre-processed sequence and epigenomic features from the context window are passed to two separate feature extraction sub-networks composed of dense layers with ReLU activation. The sequence and epigenomic feature embeddings are then merged by concatenation and fed into the transformer encoder modules, which incorporate the long-range context information by learning the pairwise dependencies between all the input loci.

The transformer encoder module is adapted from Vaswani et al. (2017). The architecture is described in Fig. 1C, which consists of one multi-head attention layer and one position-wide feed-forward layer. The multi-head attention layer trains multiple attention heads independently to attend to different parts of the sequence.


(1) $$\text{Multihead}(Q,K,V)=\text{Concat}({\text{head}}_{1},\ldots ,{\text{head}}_{h})W^{O}$$

(2) $${\text{where head}}_{i}=\text{Attention}(QW_{i}^{Q},KW_{i}^{K},VW_{i}^{V})$$

Note that *W^Q^*, *W^K^*, *W^V^*, and *W^O^* are projection matrices with trainable parameters. The attention mechanism uses the scaled dot product of the key and query to calculate the relevance between two loci. Specifically, it is defined as:


(3) $$\text{Attention}(Q,K,V)=\text{softmax}\left(\frac{QK^{T}}{\sqrt{d_{k}}}\right)V$$

The raw attention weight *QK^T^* is normalized by the factor $\sqrt{d_{k}}$, which is the dimension of the keys, and a softmax function. In addition, layer normalization is applied after each layer for better generalization. Residual connections allow the gradient to flow directly through the layers to mitigate the vanishing gradients problem. A dense layer with the Tanh activation function is applied to the output embedding of the transformer to predict the TSA-seq signals. To incorporate the positional information of the genomic bins, we adapted the relative positional embedding method introduced in TransformerXL (Dai et al. 2019).

For cross-cell-type predictions, we attached a domain adaptation module (Ganin and Lempitsky 2015) to prevent the model from overfitting on the training set (Fig. 1B). This domain adaptation module is an additional classifier that identifies the source cell types of the embeddings. The forward pass leverages the cell-type-specific information within the embeddings to predict the source cell type. The backward pass contains a gradient reversal layer that reverses the gradient flowing back into the transformer. As a result, the model will maximize the loss for cell type prediction, thus discouraging the learning of any cell-type-specific features that can be used to distinguish between the cell types.

### 2.3 Model training and evaluation

For all experiments, we adopt a cross-chromosome evaluation approach, where odd-numbered chromosomes are used for training and validation, and even-numbered chromosomes are held out for testing. To search for the optimal hyperparameters, we conducted cross-validation by holding out one odd-numbered chromosome as a validation set for each fold. The models were trained by minimizing the mean squared error (MSE) for the prediction of TSA-seq signals. Learning rate warmup and decay were defined in a similar way as described in Vaswani et al. (2017). We recorded the validation loss for each epoch and used the model parameter which yields the minimum validation metrics as the final model for downstream evaluation on the testing sets and model interpretation.

To evaluate the performance and the generalizability of the model, we ran two types of evaluations: single-cell-type prediction and cross-cell-type prediction on four human cell lines (K562, H1, HCT116, HFFc6). For single-cell-type prediction, the model is trained on the sequence and epigenomic features in one single cell type. For cross-cell-type prediction, one cell type is held out from the training to estimate the performance of our model on an unseen cell type in the real world. The model is trained on the odd-numbered chromosomes of three cell types and tested on the even-numbered chromosomes of the held-out cell type.

As a performance comparison, we also trained and evaluated the following four machine learning models. XGBoost is an optimized library for gradient tree boosting (GTB), which is a simple but powerful decision tree-based machine learning model (Chen and Guestrin 2016). Dense neural networks (DNN) consist of fully connected layers that perform nonlinear transformations to the input. XGBoost and DNN predict the signal without considering any context information. CNNs contain convolutional layers that are able to incorporate local information from neighboring bins. They have been extensively used in sequence-based predictive models, such as DeepSEA (Zhou and Troyanskaya 2015). However, different from the previous methods that predict the signal from raw DNA sequences, CNN is applied on the pre-engineered sequence features instead of the one-hot encoding of sequences. In addition, we included dilated CNNs, which boost the performance of CNNs by increasing the size of the context that can be incorporated. To ensure a fair comparison, we have utilized the same input features and context length for the CNN and dilated CNN models as we have for UNADON. The machine learning algorithms were implemented using the scikit-learn library (Pedregosa et al. 2011). The neural network-based models were implemented using PyTorch (Paszke et al. 2019).

For cross-cell-type predictions, we evaluated an additional baseline model inspired by Schreiber *et al.* (2020). Considering that the spatial positioning of chromosomes shares some similarities across cell types, it is reasonable to use the average signals of existing cell types as an estimation for an unseen cell type.

### 2.4 Interpretation of neural network

To reveal the contribution of the input features to the final predictions, we applied integrated gradients (Sundararajan et al. 2017, Kokhlikyan et al. 2020) to derive the locus-specific importance scores for sequence and epigenomic signals separately. Integrated gradients is a gradient-based feature attribution method that calculates the importance scores based on the gradient of the output compared to the input features. Intuitively, if a small deviation from the baseline causes a big change in the output, the feature should be important for the prediction. Integrated gradients considers a straight-line path between the input and the baseline. The importance score is defined by accumulating the gradient of all points on this path. We used the vector of all zeros as the baseline. Since importance scores computed by integrated gradients can be either positive or negative based on the effect of the features on the predictions, we took the absolute value of the importance scores as feature contribution. To quantify the total contribution of DNA sequence features, we aggregated the contribution from all dimensions by summing up the importance scores.

### 2.5 Data collection and preprocessing

We collected the genome-wide mapping of cytological distance to nuclear bodies from TSA-seq data in four human cell lines (K562, H1, HCT116, HFFc6) (Dekker et al. 2017,   Chen et al. 2018, Zhang et al. 2020). We followed the same normalization process as discussed in Zhang et al. (2020), which converted the normalized read counts to the TSA-seq enrichment scores. We calculated the TSA-seq scores for non-overlapping 25 kb genomic bins and scaled the signal between –1 and 1. To alleviate the effect of technical noise, we smoothed the TSA-seq signal using a Hanning window of size 21. Higher signals represent a closer distance to a specific type of nuclear body.

We used the human reference genome GRCh38. Genomic regions with low mappability, such as the centromere region, were removed. To reduce the dimensionality of DNA sequences, we use k-mer frequency to represent the sequences. We merged k-mers with their reverse complements to consider both the forward and the reverse strands of the DNA. We concatenated the k-mer frequencies for *k *=* *5 and *k *=* *6 and applied PCA to reduce the dimension of the k-mer frequency vector to 20.

To capture the cell-type-specific chromatin spatial positioning, we incorporated the widely available epigenomic features. Specifically, we aggregated chromatin accessibility and eight histone modifications (H2A.Z, H3K4me1, H3K4me2, H3K4me3, H3K9me3, H3K27me3, H3K27ac, H3K36me3) from different assays and database (Consortium et al. 2012, Dekker et al. 2017, Janssens et al. 2018), as summarized in Supplementary Table 1. We used the peaks of the signal called by the ENCODE peak calling pipeline (Consortium et al. 2012) to represent the epigenomic signals within a 25 kb genomic region. For each individual region, we count the number of bases underlying the signal peaks and normalize it by the length of the region. To account for potential technology-related bias, we further normalize the peak occurrence by the overall average peak frequency on the whole genome.

## 3 Results

### 3.1 UNADON accurately predicts chromatin spatial positioning relative to nuclear bodies in individual cell type

**Figure 2.**
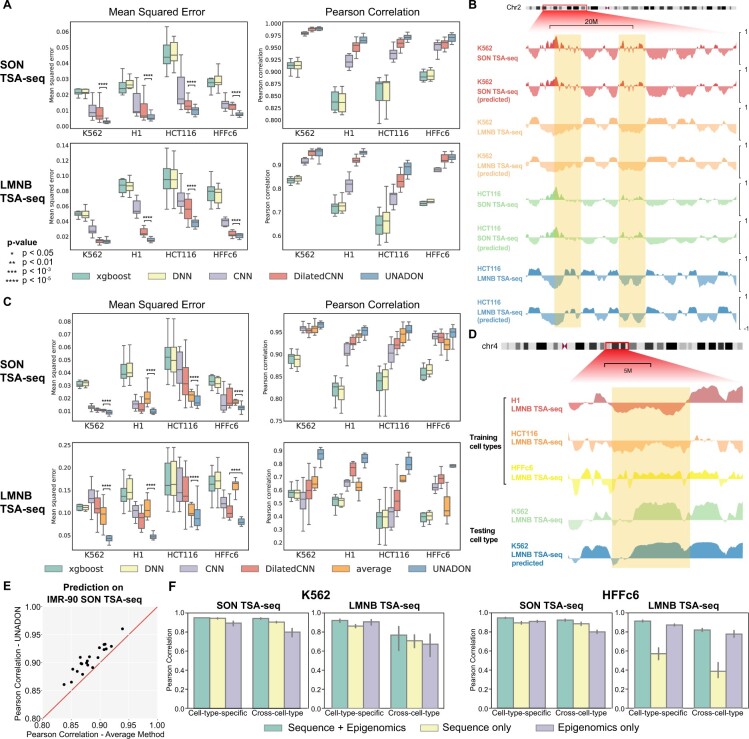
Performance evaluation and comparison for the prediction of SON and LMNB TSA-seq signal using different baseline methods in K562, H1, HCT116, and HFFc6. The boxplots show the MSE and Pearson correlation evaluated across the even-numbered chromosomes, which are held out for testing. (A) Performance of single-cell-type models measured by MSE and Pearson correlation in individual cell types. The *P*-values for performance improvement are calculated based on the squared error per genomic locus. (B) An illustration of actual and predicted TSA-seq in K562 and HCT116. UNADON is able to make accurate predictions for both SON TSA-seq and LMNB TSA-seq, as shown in the highlighted regions. (C) Performance of cross-cell-type models measured by MSE and Pearson correlation. The models were trained on the odd-numbered chromosomes of three cell types and tested on the even-numbered chromosomes of one cell type. The *x*-axis labels are the testing cell types. (D) An illustration of cross-cell-type prediction results. The model accurately predicts the highlighted cell-type-specific patterns of K562 LMNB TSA-seq that do not appear in any of the training cell types. (E) Prediction performance on external IMR-90 SON TSA-seq. The scatterplot shows the performance comparison of the average method and UNADON measured by Pearson correlation. The dots represent the 22 autosomes in IMR-90. (F) Ablation studies to demonstrate the importance of both sequence and epigenomic features for the prediction.

We applied UNADON to predict the spatial positioning of chromosomes relative to nuclear speckle (measured by SON TSA-seq) and nuclear lamina (measured by LMNB TSA-seq) in four human cell lines (K562, H1, HCT116, and HFFc6) (Fig. 2A). Examples of the comparison of the predicted signals and the actual TSA-seq tracks are shown in Fig. 2B. The highlighted region illustrates that UNADON accurately predicts the cell-type-specific patterns of the spatial positioning of chromosomes relative to specific nuclear bodies. Importantly, even the small structures, such as the tiny peaks and valleys smaller than 1 Mb, are predicted correctly.

We compared the performance of UNADON with four commonly used baseline methods: XGBoost, DNNs, CNNs, and Dilated CNNs (see Section 2) in terms of MSE and Pearson correlation coefficient (PCC). We found that UNADON consistently outperforms the baseline models in all cell types and nuclear bodies (Fig. 2A). Specifically, UNADON reaches a median Pearson correlation of 0.97 on SON TSA-seq and 0.92 on LMNB TSA-seq across all four cell types, which is significantly higher than XGBoost and DNNs with no context information. UNADON also outperforms the basic CNN in all prediction tasks, suggesting that long-range dependencies are important for the prediction. Remarkably, UNADON achieves better prediction performance than the state-of-the-art dilated CNN, further demonstrating the strength of the attention mechanism in capturing the relationship between genomic features and spatial positioning. Note that the prediction performance of UNADON differs across the cell types, especially on LMNB TSA-seq. The PCC in HCT116 LMNB TSA-seq is only 0.88, as compared with 0.95 in K562 LMNB TSA-seq. The discrepancy indicates that the cell types may contain distinct sequences and epigenomic features for large-scale chromosome positioning, which increases the difficulty of cross-cell-type prediction.

Overall, these evaluations confirm the advantage of UNADON over the baseline methods for predicting the spatial positioning of chromosomes in individual cell types.

### 3.2 UNADON can predict chromatin spatial positioning in unseen cell types

To examine the ability of UNADON to generalize to unseen cell types, we conducted cross-cell-type evaluations by holding out one cell type for testing and training the models on the remaining three cell types. We included an additional baseline method, named ‘average’, which simply utilizes the average TSA-seq in training cell types as the prediction. The average method also serves as a measure of how similar the spatial positioning is in the testing cell type compared with that in the training cell types. Similar to the single-cell-type models, we evaluated the performance by calculating the mean squared error and Pearson correlation. The advantage of UNADON over the baseline models becomes more pronounced in the cross-cell-type setting, as is shown in Fig. 2C. SON TSA-seq is highly conserved among the cell types, as is revealed by the strong performance of the average method (median PCC 0.94) and the conserved example regions in Fig. 2B. As a result, all baseline models reach a PCC above 0.8 on predicting cross-cell-type SON TSA-seq, where UNADON is the highest in all evaluations (median PCC 0.956).

LMNB TSA-seq is more variable across the cell types, as indicated by poor PCC (0.62) of the average method and the highly variable example regions in Fig. 2B. Regardless, UNADON, with a median PCC between 0.785 (HFFc6) and 0.875 (K562), significantly outperforms all baseline methods, including CNNs and dilated CNNs that incorporate context information. The result suggests that the transformer modules and the domain adaptation design are crucial for the generalizability of the models. In addition, we provided an example visualization of the K562 LMNB TSA-seq signals predicted by the cross-cell-type UNADON. We also included the tracks from the training cell types as references. Based on Fig. 2D, it is clear that UNADON is capable of predicting the distinct patterns of K562 LMNB TSA-seq, further confirming that UNADON is indeed learning the fundamental connections between the spatial positioning and the genomic features.

To further demonstrate the ability of UNADON to infer TSA-seq on a new cell type, we conducted an evaluation using an external IMR-90 SON TSA-seq dataset from a different lab (Alexander et al. 2021). We reprocessed the raw data based on the TSA-seq preprocessing workflow (see Section 2). UNADON achieves a mean Pearson correlation of 0.91 across all 22 chromosomes in IMR-90, which is consistently better than the performance of the average method, as is shown in Fig. 2E. The evaluation of UNADON on the external dataset provides additional validation of its ability to accurately predict TSA-seq in unseen cell types, supporting its practical utility and potential for inferring chromatin spatial positioning in new cell types in the absence of TSA-seq data.

In addition, to probe the importance of different input features to the prediction performance, we conducted ablation studies by removing either sequence or epigenomic features from the input (Fig. 2F). For SON TSA-seq, sequence features alone are sufficient to reach a high prediction performance, suggesting the crucial role of sequence features in determining chromatin positioning toward nuclear speckles. The incorporation of epigenomic features slightly boosts the performance by providing additional information of the chromatin states. For LMNB TSA-seq, the result varies depending on the cell type. In K562, the sequence-only models attain a moderate prediction performance. However, in HFFc6, sequence features are significantly less predictive of the LMNB TSA-seq. In this case, epigenomic features are necessary for the accurate prediction of the spatial positioning of chromatin. These results suggest that different cell types may utilize distinct underlying mechanisms for targeting the chromatin toward nuclear lamina. Therefore, both sequence and epigenomic features play critical roles in achieving the high prediction performance of UNADON.

Taken together, the cross-cell-type evaluations demonstrate that UNADON can still perform well in predicting chromatin spatial positioning in unseen cell types.

### 3.3 UNADON uncovers the genome-wide contribution of sequence and epigenomic features

**Figure 3.**
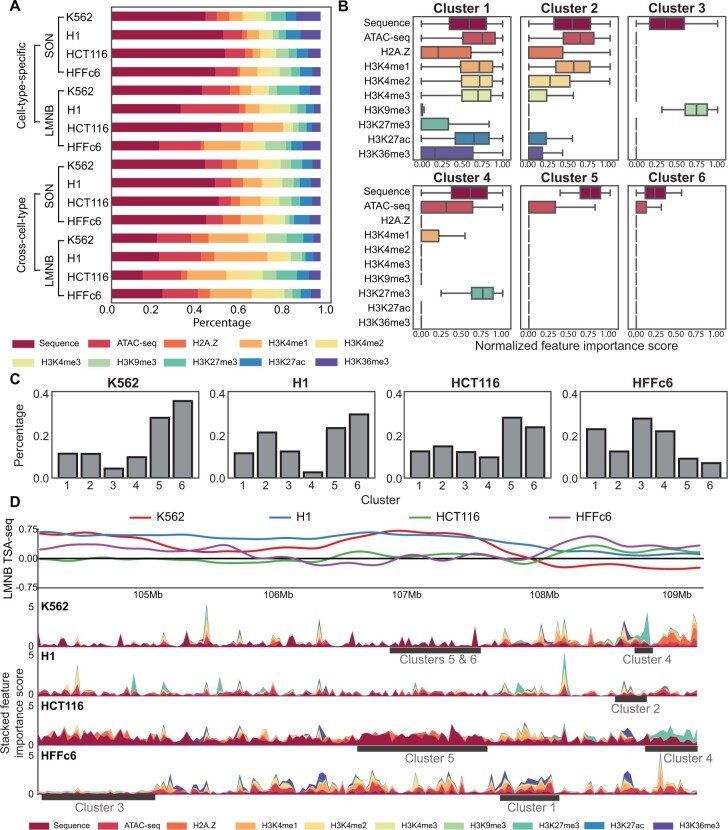
The feature importance scores computed by integrated gradients delineate the genome-wide contribution of DNA sequences and epigenomic signals from LMNB TSA-seq across all four cell types. (A) Percentage contribution of sequence and epigenomic features across all experimental setups. (B) Six distinct patterns of sequence and epigenomic contributions are identified by k-means clustering, each with a unique distribution of the normalized importance scores. (C) Proportion of each pattern of feature contribution in the four cell types. (D) Example of the distribution of feature importance score on chr4 across the four cell types. The top track displays the LMNB TSA-seq signals. The bottom tracks are stacked plots of feature importance scores. The black bars underneath the important score tracks highlight the representative regions for each cluster of patterns.

We next applied integrated gradients to investigate the contribution of sequence and epigenomic features to the prediction of both SON TSA-seq and LMNB TSA-seq across the four cell types. We first computed the mean importance score of each individual feature by averaging over all the genomic bins on the test chromosomes (Fig. 3A). We found that overall the DNA sequence is the most important feature for prediction, followed by ATAC-seq and H3K4me1. The distribution of feature importance varies drastically across different cell types and target nuclear bodies. The contribution of sequence in predicting LMNB TSA-seq is comparatively less significant compared to its contribution to predicting SON TSA-seq, suggesting that the targeting mechanisms toward nuclear lamina may be less dependent on sequence features. Another observation is that the contribution of epigenomic features in HFFc6 is greater than that in other cell types, which aligns with the findings from our ablation studies. Moreover, the contribution of epigenomic features becomes more evident in the cross-cell-type settings, since the models need cell-type-specific epigenomic information for the predictions compared to the cell-type-agnostic sequence features. This analysis shows a quantitative assessment of the role of sequence and epigenomic features in modulating chromatin spatial positioning toward nuclear bodies.

To gain insight into how different features collectively contribute to the prediction, we performed k-means clustering to identify patterns of the importance score distribution. Given that the role of sequence and epigenomic features are more intertwined in the prediction of LMNB TSA-seq, we focus on the contribution scores derived from LMNB TSA-seq across four cell types. The optimal number of clusters was determined through silhouette analysis, resulting in the identification of six distinct patterns (Fig. 3B). The proportion taken up by each cluster is reported in Fig. 3C. Cluster 1 has high sequence and epigenomic contributions from active histone marks, including H3K4me1, H3K4me2, H3k4me3, and H3K27ac. Cluster 2 is characterized by the high contribution from ATAC-seq and H3K4me1, along with low to moderate contributions from H2A.Z and H3K4me2. Cluster 3 has a unique high contribution from H3K9me3. Cluster 4 is distinguished by a high contribution from H3K27me3. Clusters 5 and 6 represent genomic regions where only DNA sequence is informative for the prediction. Interestingly, cluster with low sequence and high epigenomic contribution is not detected, further emphasizing the essential role of sequence features.

Moreover, we show the distribution of feature importance scores in Fig. 3D in a 5 Mb region to demonstrate how various cell types use different patterns to predict cell-type-specific LMNB TSA-seq signals. Representative regions for each cluster are annotated for each cluster at the bottom. We observed that clusters 1 and 2 tend to be smaller, marked by the sharp peaks on the plot. Clusters 5 and 6, with high and low sequence-only contributions, respectively, are usually intertwined with each other, spanning a large region of the genome.

Overall, these results provide new insight into the underlying targeting mechanism of large-scale chromatin toward nuclear bodies, which will be further analyzed in the next section.

### 3.4 UNADON identifies the potential sequence and epigenomic determinants for chromatin localization

**Figure 4.**
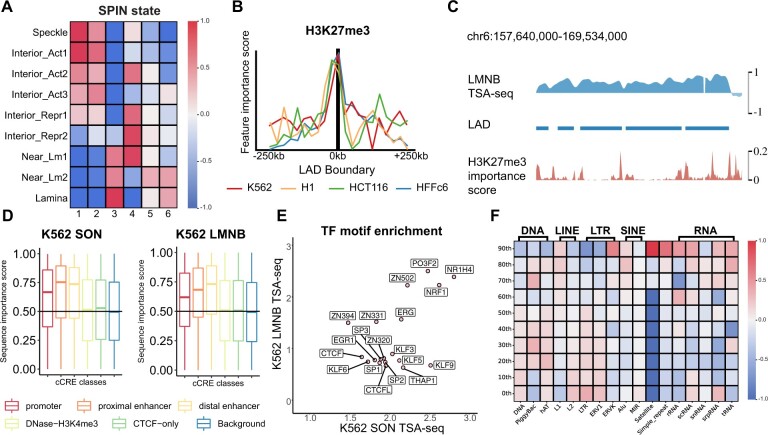
Analysis of the feature importance scores reveals the important sequence element and epigenomic features for chromatin spatial positioning relative to nuclear bodies. (A) The comparison of the pattern clusters with SPIN states (Wang et al. 2021). The colors represent the log2 fold change of the percentage of the SPIN states in a specific cluster relative to the randomly sampled background set. (B) The distribution of H3K27me3 importance score near the LAD boundaries. (C) Example of the correlation between LAD boundaries and H3K27me3 importance score on chr6: 157 640 000–169 534 000. (D) The sequence importance scores in the top 5% genomic regions with the highest enrichment of the cCREs annotated by SCREEN (Moore et al. 2020). The randomly sampled background set is included as a reference. (E) The TF motif enrichment log2 fold change of the top 10% most important genomic regions for the prediction of K562 SON TSA-seq and K562 LMNB TSA-seq. (F) The repetitive element enrichment fold change of genomic regions with different sequence importance levels. The genomic regions in the test chromosomes are divided into 10 groups based on the importance scores. The repetitive element families are labeled on the top.

Next, we sought to explore why the sequences and histone modifications identified in the previous section are important for chromatin spatial positioning. We first inspected the genome-wide distribution of each pattern by comparing it with the SPIN states (Wang et al. 2021), which provides a genome-wide annotation for nuclear compartmentalization by integrating TSA-seq, DamID, and Hi-C (Fig. 4A), in all four cell types. The SPIN states include Speckle, Interior Active 1-3, Interior Repressive 1-3, Near Lamina 1-2, and Lamina. We observed that clusters 1 and 2, with significant joint contributions from multiple histone marks, are predominantly located in the Speckle and Interior Active regions, which are found to have low LMNB TSA-seq signals (Wang et al. 2021). In other words, regions with these two patterns of feature contribution are generally distant from nuclear lamina, which may not be directly involved in the targeting mechanisms but instead informative in predicting regions with low TSA-seq signals. Therefore, clusters 1 and 2 suggest that the model leverages the pattern of active histone marks such as H3K4me1 to predict the low LMNB TSA-seq regions, which is supported by the previous findings that the non-lamina-associated domains (LADs) have higher enrichment of active histone marks (Van Steensel and Belmont 2017). Cluster 3, characterized by the high contribution of H3K9me3 and sequence features, is located exclusively in the Lamina and Near Lamina regions, consistent with the earlier findings that LADs are enriched with H3K9me3 (Briand and Collas 2020). Cluster 4, consisting of regions with high contribution of H3K27me3 and sequence features, is located in both interior active and repressive states. Recent studies (Harr et al. 2015, Chen et al. 2018) revealed that H3K27me3 is highly enriched in the border of LADs and is potentially involved in LAD formation. Thus, we hypothesize that the model relies on H3K27me3 to predict the proximity to nuclear lamina, especially the sharp increase and decrease that indicates the formation of the LADs. To test this hypothesis, we assessed the importance score of H3K27me3 near the LAD boundaries (Fig. 4B). For all cell types, we detected a sharp peak in H3K27me3 feature importance at the LAD boundary, which is not observed for other histone marks nor in H3K27me3 for SON TSA-seq predictions. Figure 4C illustrates the clear correspondence between the H3K27me3 importance score peaks and the LAD boundaries. Together, these analyses confirm the role of H3K27me3 in predicting the TSA-seq signals near the LAD boundaries. Clusters 5 and 6, where only sequence features are important for prediction, are located preferentially in repressive states, including Near Lamina 1-2 and Lamina, indicating that the models rely on sequence features to predict regions lacking histone marks.

To uncover the sequence features predictive of chromosome positioning relative to nuclear lamina, we conducted a sequence composition analysis to explore what sequence elements are significantly enriched in the high-importance regions. Particularly, we compared the sequence importance score with existing sequence annotations, including candidate cis-regulatory elements (cCRE), transcription factor (TF)-binding motif, and repetitive elements. We randomly sampled a background set of genomic regions for the purpose of comparison. To explore whether regulatory elements are predictive of the spatial positioning of chromatin, we assessed the importance score for the top 5% genomic regions with the highest enrichment of the cCREs annotated by SCREEN (Moore et al. 2020). We observed that regions enriched in promoters and enhancers are assigned considerably higher importance scores than background regions (Fig. 4D), suggesting that promoters and enhancers are potentially involved in large-scale chromatin spatial positioning. Next, we performed TF motif enrichment analysis based on 401 motifs in the human genome collected in HOCOMOCO v11 CORE (Kulakovskiy et al. 2018). We utilized FIMO (Grant et al. 2011) to scan through the entire genome and counted the frequency of motif (*q*-value <0.05) within each 25 kb bin. We divided the genomic regions on the test chromosomes into ten groups based on the importance scores and calculated the motif enrichment for each group. Figure 4E shows the top motifs with the highest enrichment in the most important group for the prediction of K562 SON TSA-seq and K562 LMNB TSA-seq. A large number of TFs belong to the Sp/KLF family. Notably, we found that CTCF is highly enriched in the important regions, which matches the previous findings on the role of CTCF in modulating LAD boundaries (Harr et al. 2015). In addition, we compared the sequence importance score with repetitive elements (Fig. 4F). The important regions have a higher abundance of ERVK, which is a long tandem repeat, and a moderate enrichment of the small RNAs (scRNA and srpRNA). Consistent with previous reports on the association between LADs and LINE, LINE element L1 is slightly enriched in the most predictive loci (Meuleman et al. 2013). We found that the satellite DNA is exclusively enriched in the most important regions, suggesting its highly predictive role for LMNB TSA-seq. We repeated the same analysis for the other three cell types, and we observed that the identified important sequence elements are highly conserved across cell types.

Together, these analyses of feature importance scores and specific sequence properties further identified potential sequence and epigenomic determinants for chromatin spatial positioning to nuclear bodies.

## 4 Discussion

In this work, we developed UNADON as a novel deep learning-based method to predict the spatial positioning of the chromosomes relative to functional nuclear bodies and explore the potential sequence and epigenomic determinants for modulating such spatial positioning in different cell types. UNADON takes advantage of the state-of-the-art transformer model and the multi-modal design to incorporate multi-omic data across a large context. With the distinct neural architecture design, UNADON demonstrates strong prediction performance on individual cell types and generalizes accurately to unseen cell types. Extensive analysis of the feature importance score reveals important regions that are consistent with previous experimental findings and further proposes potential mechanisms for the targeting of large-scale chromatin toward nuclear bodies. Overall, UNADON establishes a novel framework for the prediction and interpretation of chromosome positioning relative to nuclear bodies, which can be extended to any large-scale sequence-based predictive modeling and analysis.

There are a number of directions to further improve UNADON. First, the attention mechanism inside the transformer layers can be further used to enhance interpretability. Attention weights have been widely adopted to probe pairwise dependencies in sequence data (Vaswani et al. 2017). However, the utility of attention as explanations is currently a subject of ongoing debate. It would be worthwhile to investigate whether attention mechanisms have the potential to provide novel insights into the interactions between different genomic regions in DNA sequences. Second, the feature importance scores computed by the post-hoc methods detect only correlations, not causation. As a result, the high-importance regions, such as clusters 1 and 2 that we identified in this work, are predictive of the LMNB TSA-seq signals but not necessarily indicative of the targeting mechanism toward the nuclear lamina. In future work, perturbation-based methods can be leveraged to study the causal relationship between chromosome positioning and the sequence and epigenomic features. These results can in turn enhance the predictive models. Finally, the correlation between spatial positioning relative to different nuclear bodies and 3D genome features has been reported (Chen et al. 2018, Belmont 2022), which makes it intriguing to build an integrative predictive model that combines spatial positioning of chromosomes and chromatin interactions. We envision that the future versions of UNADON will connect 1D genome features, including both sequence features and epigenomic properties, with 3D chromatin structure and positioning in a more cohesive and interpretable way.

## Supplementary Material

btad246_Supplementary_Data
