# Supplementary material for: UNADON: transformer-based model to predict genome-wide chromosome spatial position
Source: Bioinformatics. 2023 Jun 30;39(Suppl 1):i553–62. doi: 10.1093/bioinformatics/btad246 (PMC10311299; doi:10.1093/bioinformatics/btad246)
Supplement: btad246_Supplementary_Data [file btad246_supplementary_data.pdf]

UNADON: Transformer-based model to predict genome-wide chromosome spatial position

(SUPPLEMENTAL INFORMATION)

A Supplementary table

Data	Source	Identifier
**K562**		
ATAC-seq	ENCODE	ENCSR868FGK
H2A.Z ChIP-seq	ENCODE	ENCSR000APC
H3K4me1 ChIP-seq	ENCODE	ENCSR000EWC
H3K4me2 ChIP-seq	ENCODE	ENCSR000AKT
H3K4me3 ChIP-seq	ENCODE	ENCSR668LDD
H3K9me3 ChIP-seq	ENCODE	ENCSR000APE
H3K27me3 ChIP-seq	ENCODE	ENCSR000EWB
H3K27ac ChIP-seq	ENCODE	ENCSR000AKP
H3K36me3 ChIP-seq	ENCODE	ENCSR000AKR
**H1**		
ATAC-seq	4DN data portal	4DNESLMCRW2C
H2A.Z ChIP-seq	ENCODE	ENCSR571IIS
H3K4me1 ChIP-seq	ENCODE	ENCSR271TFS
H3K4me2 ChIP-seq	ENCODE	ENCSR322MEI
H3K4me3 ChIP-seq	ENCODE	ENCSR443YAS
H3K9me3 ChIP-seq	ENCODE	ENCSR883AQJ
H3K27me3 ChIP-seq	ENCODE	ENCSR928HYM
H3K27ac ChIP-seq	ENCODE	ENCSR880SUY
H3K36me3 ChIP-seq	ENCODE	ENCSR476KTK
**HCT116**		
ATAC-seq	ENCODE	ENCSR872WGW
H2A.Z ChIP-seq	ENCODE	ENCSR227XNT
H3K4me1 ChIP-seq	ENCODE	ENCSR161MXP
H3K4me2 ChIP-seq	ENCODE	ENCSR794ULT
H3K4me3 ChIP-seq	ENCODE	ENCSR333OPW
H3K9me3 ChIP-seq	ENCODE	ENCSR179BUC
H3K27me3 ChIP-seq	ENCODE	ENCSR810BDB
H3K27ac ChIP-seq	ENCODE	ENCSR661KMA
H3K36me3 ChIP-seq	ENCODE	ENCSR091QXP
**HFFc6**		
ATAC-seq	4DN data portal	4DNESMBA9T3L
H2A.Z CUT&RUN	4DN data portal	4DNESIBPKCJK
H3K4me1 Mint-ChIP-seq	ENCODE	ENCSR340XKM
H3K4me2 CUT&RUN	4DN data portal	4DNESWK53WP1
H3K4me3 Mint-ChIP-seq	ENCODE	ENCSR639PCR
H3K9me3 Mint-ChIP-seq	ENCODE	ENCSR938NXC
H3K27me3 Mint-ChIP-seq	ENCODE	ENCSR129TUY
H3K27ac Mint-ChIP-seq	ENCODE	ENCSR510VXV
H3K36me3 Mint-ChIP-seq	ENCODE	ENCSR519CMW
**IMR-90**		
ATAC-seq	ENCODE	ENCSR200OML
H2A.Z ChIP-seq	ENCODE	ENCSR124DYB
H3K4me1 ChIP-seq	ENCODE	ENCSR831JSP
H3K4me2 ChIP-seq	ENCODE	ENCSR672XZZ
H3K4me3 ChIP-seq	ENCODE	ENCSR087PFU
H3K9me3 ChIP-seq	ENCODE	ENCSR055ZZY
H3K27me3 ChIP-seq	ENCODE	ENCSR431UUY
H3K27ac ChIP-seq	ENCODE	ENCSR002YRE
H3K36me3 ChIP-seq	ENCODE	ENCSR437ORF

Table S1: Dataset used in this paper
